# Wear resistance and fracture strength of BioFlx, zirconia and stainless steel crowns for primary molars after thermomechanical aging: an in vitro comparative study

**DOI:** 10.1186/s12903-026-08912-4

**Published:** 2026-06-23

**Authors:** Alp Abidin Ateşçi, Dilek Özge Yılmaz, Berk Gergit, Münevver Çoruh Kılıç, Hüseyin Şimşek

**Affiliations:** 1 Department of Pediatric Dentistry, School of Dentistry, Istanbul Beykent University, Büyükçekmece, Istanbul Türkiye; 2 Department of Pediatric Dentistry, School of Dentistry, Atatürk University, Erzurum, Türkiye; 3 Department of Pediatric Dentistry, School of Dentistry, Biruni University, Istanbul, Türkiye; 4 Department of Pediatric Dentistry, School of Dentistry, Ordu University, Ordu, Türkiye

**Keywords:** Pediatric crown, Primary molar, BioFlx, Zirconia crown, Stainless steel crown, Occlusal wear, Fracture resistance, Thermomechanical aging

## Abstract

**Background:**

Full-coverage restorations are often required for primary molars with extensive carious destruction. Stainless steel crowns remain the long-standing reference, while prefabricated zirconia and polymer-based crowns have become tooth-colored alternatives. Direct comparisons under a single thermomechanical protocol, with separate wear and fracture cohorts, remain limited. This in vitro study quantified post-aging occlusal wear and compressive fracture load in four prefabricated crown systems for mandibular second primary molars.

**Methods:**

Eighty crowns were tested, with 10 specimens per material for each outcome: two prefabricated zirconia crowns, one polymer-based crown, and one stainless steel crown. Identical resin dies were 3D-printed and all crowns were luted with glass-ionomer cement. All specimens underwent 10,000 thermal cycles between 5 °C and 55 °C. The wear cohort underwent 150,000 chewing cycles at 50 N and 1.2 Hz, and wear was scored on a 0–3 scale by two blinded examiners. The fracture cohort was loaded to failure. Wear scores were compared using Kruskal–Wallis and Dunn–Bonferroni tests; fracture loads were analyzed using one-way ANOVA and Tukey HSD.

**Results:**

Mean wear scores were 1.1 ± 0.3 for NuSmile, 1.2 ± 0.4 for ProfZr, 1.7 ± 0.4 for SSC, and 2.2 ± 0.4 for BioFlx. The zirconia groups did not differ significantly (*p* = 0.68), whereas both showed lower wear scores than SSC (*p* = 0.04) and BioFlx (*p* = 0.01). SSC also showed lower wear scores than BioFlx (*p* = 0.03). Mean fracture loads were 3148.4 ± 643.4 N for BioFlx, 2703.4 ± 243.1 N for SSC, 1076.1 ± 186.9 N for NuSmile, and 565.5 ± 159.6 N for ProfZr (*p* < 0.001). BioFlx and SSC did not differ significantly (*p* = 0.093), while both exceeded the zirconia groups (*p* < 0.001). NuSmile exceeded ProfZr (*p* = 0.043).

**Conclusions:**

Under this in vitro model, zirconia crowns showed the lowest wear scores, whereas BioFlx and SSC showed the highest fracture loads. Each material demonstrated a distinct mechanical profile. Laboratory rankings alone should not guide clinical recommendations; long-term clinical studies are needed.

**Supplementary Information:**

The online version contains supplementary material available at https://doi.org/10.1186/s12903-026-08912-4.

## Background

Dental caries remains a frequent chronic disease in early childhood, and the primary molars are particularly affected because of their thin enamel, broad proximal contacts, and prolonged functional period before exfoliation [1]. When the residual coronal tissue is insufficient to retain an intracoronal restoration, a full-coverage crown is indicated. Preformed stainless steel crowns (SSCs) have decades of clinical track record in pediatric dentistry, with high survival rates also under the modified Hall technique [2, 3]. However, their metallic appearance has increased the demand for tooth-colored alternatives [4, 5].

Prefabricated zirconia crowns were introduced to address this esthetic limitation. Several clinical reports describe good gingival adaptation, parental acceptance and three-year survival rates of 76–94% [6–8]. However, zirconia crowns generally require greater tooth reduction than SSCs and offer less flexibility for chairside adjustment, marginal adaptation, and occlusal modification [9, 10].

More recently, prefabricated polymer-based crowns (BioFlx) have been introduced as tooth-colored options with a lower elastic modulus and simplified preparation requirements. The material contains neither a metallic substructure nor Bis-GMA, and its relatively low elastic modulus has been proposed to affect load distribution under occlusal forces. Early in vitro studies indicate that such polymer crowns can sustain loads relevant to pediatric mastication while requiring less tooth reduction than zirconia [11, 12].

Preclinical studies commonly use thermocycling and chewing simulation to reproduce selected aspects of intraoral aging under controlled conditions, although these protocols cannot fully represent the clinical environment [13, 14]. Kessler et al. showed that crown material and cementation method jointly modulate wear and fracture resistance in primary molar crowns [15].

Comparative evidence for BioFlx remains limited. A finite-element analysis comparing BioFlx, zirconia and SSC reported lower simulated stresses in the BioFlx models under axial and oblique loading, while also emphasizing the need for empirical aging data [16]. A subsequent in vitro report placed BioFlx between SSC and zirconia for fracture load, but the available dataset was small and brand-dependent [17]. To our knowledge, no previous study has compared BioFlx with two zirconia systems and SSC under a single standardized thermomechanical protocol with independent specimen cohorts for wear and fracture, a design that avoids exposing fracture specimens to the additional fatigue generated during wear testing.

The objective of the present study was to quantify, in mandibular second primary molar crowns, (i) post-aging occlusal wear and (ii) compressive fracture load for two zirconia systems (NuSmile, ProfZr), one polymer-based crown system (BioFlx), and one stainless steel crown system (Kids Crown SSC). The null hypothesis was that none of the four materials would differ statistically from the others for either outcome.

## Methods

### Sample size calculation and grouping

Sample size was estimated a priori using G*Power 3.1 (Faul, Erdfelder, Lang, & Buchner; Düsseldorf, Germany) under a one-way fixed-effects ANOVA with α = 0.05, power (1 − β) = 0.80 and an effect size f = 0.55. The selected effect size was based on the magnitude of fracture-resistance differences reported between SSC and zirconia crowns in the comparator study by Kessler et al. [15]. This calculation indicated a minimum of ten specimens per group. Eighty crowns were therefore obtained and assigned to four material groups, with the wear and fracture analyses run on two independent cohorts of *n* = 40 (*n* = 10 per material per outcome):

- Group 1 — prefabricated zirconia crowns (NuSmile; NuSmile, Houston, TX, USA)
- Group 2 — prefabricated zirconia crowns (ProfZr; Prof Teknoloji, Erzurum, Türkiye)
- Group 3 — prefabricated polymer-based crowns (BioFlx; Kids-e-dental, Mumbai, India)
- Group 4 — prefabricated stainless steel crowns (Kids Crown; Shinhung, Seoul, South Korea)

### Crown preparation

A virtual mandibular second primary molar preparation was generated in dental CAD software (Exocad; Exocad, Darmstadt, Germany). A single calibrated pediatric dentist (H.S.) carried out the digital design, and the resulting STL files were locked before printing to prevent unintended parameter drift. For the zirconia groups the preparation comprised 2.0 mm occlusal reduction, 1.5 mm proximal clearance and 1.0 mm circumferential reduction, with rounded internal angles and a full circumferential bevel. For the BioFlx and SSC groups occlusal reduction was reduced to 1.5 mm and the proximal and circumferential parameters were identical to those of the zirconia design.

### Die fabrication

Eighty identical resin dies replicating the master preparation were printed on a desktop 3D printer in a model resin (NextDent Model 2.0; Vertex Dental, Soesterberg, the Netherlands). Printed resin dies were used to obtain reproducible geometry and a uniform supporting substrate across all experimental groups. This standardization was considered important for isolating the effect of crown material on wear and fracture outcomes. Similar standardized resin-die approaches have been used in laboratory crown fracture studies [18]. Specimens were post-cured according to the manufacturer's instructions.

### Embedding

Each die was embedded in cylindrical blocks of auto-polymerizing methacrylate resin (Technovit 4000; Heraeus Kulzer, Wehrheim, Germany). This embedding approach was used to provide stable and reproducible support during loading, as commonly applied in in vitro fracture-resistance studies [18].

### Crown cementation

All crowns were luted with a conventional glass-ionomer cement (Fuji I; GC, Tokyo, Japan) mixed and applied according to the manufacturer's instructions. A constant axial load of 5 kg was maintained for 3 min during seating to produce reproducible cementation pressure, following the protocol of Kessler et al. [15]. Excess cement was removed and the specimens were stored in distilled water at 37 °C for 24 h to allow complete setting [13].

### Thermocycling and chewing simulation

Thermal aging was performed in agreement with ISO 11405. Each specimen completed 10,000 thermal cycles between 5 °C and 55 °C (25 s dwell, 10 s transfer) in a combined thermocycling/chewing unit (Model ACS-8; Analitik Medikal, Gaziantep, Türkiye). This protocol has been suggested to simulate approximately one year of intraoral thermal exposure for restorative materials [19]. The wear cohort received, in the same unit, an additional 150,000 chewing strokes at 50 N and 1.2 Hz, against a 4 mm steatite antagonist mounted on a vertically translating rod. Each stroke originated at the functional cusp and traversed ~ 0.3 mm towards the central fossa, simulating pediatric masticatory excursion; loading and lift-off velocities were 20 mm s⁻^1^ and 60 mm s⁻^1^ respectively, with continuous 37 °C water irrigation. The loading geometry was adapted from previously published two-body wear protocols for primary molar crowns [12, 20].

### Wear assessment

After chewing simulation each occlusal surface was inspected at × 20 with a stereomicroscope. A four-step ordinal wear index was applied: 0 = no visible wear, 1 = slight polishing marks, 2 = moderate wear facets, 3 = severe material loss [21, 22]. Two examiners (D.Ö.Y. and B.G.) independently scored coded specimen images without access to the group allocation. Inter-rater agreement was estimated using Cohen’s κ coefficient (κ = 0.87), indicating high agreement.

### Fracture resistance testing

The fracture cohort entered the test sequence after thermocycling only. Each crown–die assembly was embedded in self-cured acrylic resin, leaving 1 mm of the cervical margin exposed to mimic clinical support, and clamped vertically in a universal testing machine (Instron 2710–003; Instron, Norwood, MA, USA) fitted with a 5 kN load cell. A 7 mm diameter cylindrical stainless steel indenter was aligned perpendicular to the long axis of the crown and contacted the center of the occlusal fossa through a 0.5 mm tin-foil interposing layer that distributed load across the cusps and prevented point-contact peaks. Loading proceeded under dry, room-temperature conditions (~ 23 °C) at a crosshead speed of 1 mm min⁻^1^ until failure. Failure was registered as a sudden discontinuity in the load–displacement curve, normally accompanied by an audible crack or by separation of a crown fragment, providing a reproducible end-point. Peak load (N) was logged directly by the testing software. Each fractured assembly was then re-examined at × 20 and the failure pattern was classified as cohesive (failure inside the crown wall), adhesive (failure at the cement–crown interface) or mixed.

### Statistical analysis

Analyses were carried out using statistical software (IBM SPSS Statistics 26; IBM, Armonk, NY, USA). Descriptive data are presented as mean ± standard deviation. Distributional assumptions were checked with Shapiro–Wilk and Levene tests. Fracture loads met both assumptions (*p* > 0.05) and were compared by one-way ANOVA with Tukey HSD; η^2^ was computed to convey effect size. Wear scores violated normality and were therefore analyzed by the Kruskal–Wallis test followed by Dunn–Bonferroni post-hoc comparisons; the epsilon-squared statistic (ε^2^) was used as the effect-size estimate. Failure-mode distributions were compared by Fisher exact test. The α level was set at 0.05.

## Results

### Wear assessment

**Fig. 1:**
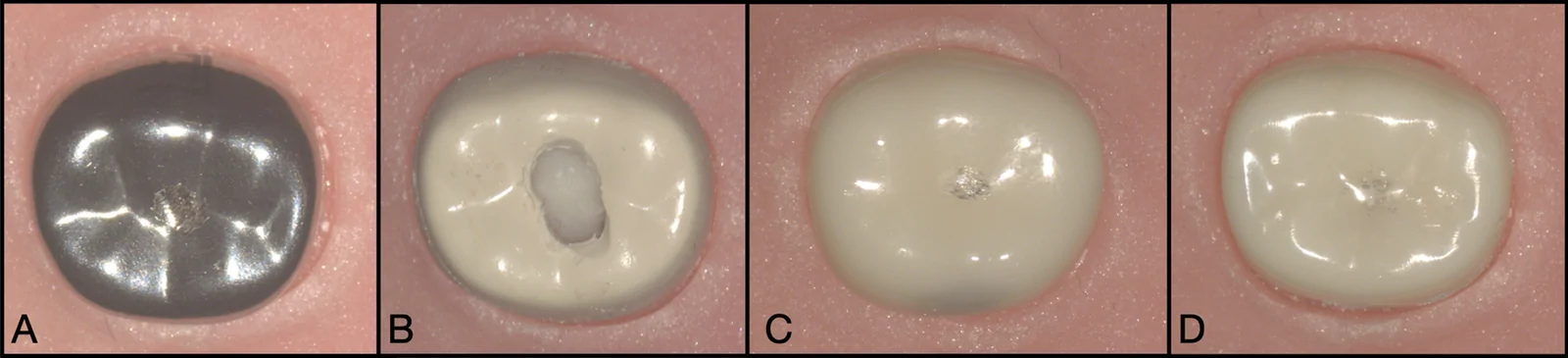
Representative occlusal surface wear patterns after 10,000 thermal cycles and 150,000 chewing cycles under 50 N (stereomicroscopy, × 20). **A** Stainless steel crown showing moderate polishing marks with limited material loss; **B** BioFlx showing pronounced wear facets and localized surface deformation; **C** ProfZr zirconia showing shallow wear traces and polished areas; **D** NuSmile zirconia showing minimal visible wear

After simulated thermomechanical aging and 150,000 chewing strokes, all crowns showed measurable occlusal wear. The Kruskal–Wallis test detected an overall difference between materials (*p* < 0.05; ε^2^ = 0.43). Mean (± SD) wear scores were 1.1 ± 0.3 for NuSmile, 1.2 ± 0.4 for ProfZr, 1.7 ± 0.4 for SSC and 2.2 ± 0.4 for BioFlx (Table 1). Dunn–Bonferroni pairwise comparisons did not detect a statistically significant difference between the two zirconia products (*p* = 0.68). Both zirconia groups had lower wear scores than SSC (*p* = 0.04) and than BioFlx (*p* = 0.01). SSC showed intermediate wear scores, with lower scores than BioFlx (*p* = 0.03) and higher scores than both zirconia groups (*p* = 0.04). Stereomicroscopic inspection (Fig. 1) corroborated this ranking: zirconia surfaces remained smooth with marginal polishing of the glaze, SSC displayed shallow wear facets, and BioFlx showed pronounced material removal and localized surface deformation.

**Table 1:** Visual wear scores (0–3 scale) of the tested crowns after thermomechanical aging and 150,000 chewing cycles

Crown type	Mean ± SD	vs NuSmile	vs ProfZr	vs SSC	vs BioFlx
NuSmile	1.1 ± 0.3	—	0.68	0.04*	0.01**
ProfZr	1.2 ± 0.4	0.68	—	0.04*	0.01**
SSC	1.7 ± 0.4	0.04*	0.04*	—	0.03*
BioFlx	2.2 ± 0.4	0.01**	0.01**	0.03*	—

Data are mean ± standard deviation. Pairwise comparisons by Dunn–Bonferroni post-hoc test following the overall Kruskal–Wallis analysis

*p < 0.05; **p < 0.01. Lower scores indicate better wear resistance

### Fracture resistance

**Fig. 2:**
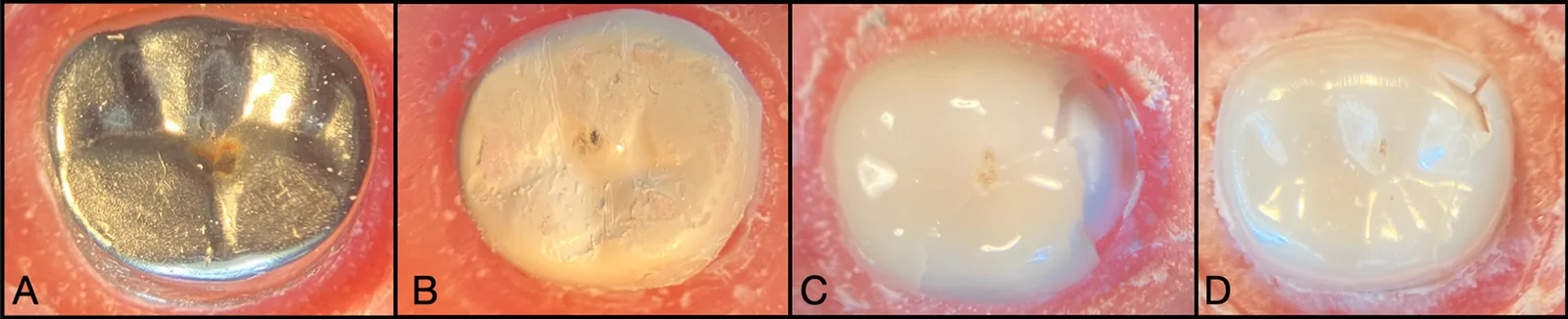
Representative post-fracture appearances after thermomechanical aging and compressive loading. **A** Stainless steel crown showing ductile surface deformation; **B** BioFlx showing localized plastic deformation and surface damage; **C** ProfZr zirconia showing marginal and cohesive cracks; **D** NuSmile zirconia showing radial crack propagation from the occlusal fossa

Fracture-load data satisfied normality (Shapiro–Wilk *p* > 0.05) and homogeneity of variance (Levene *p* > 0.05). One-way ANOVA returned an overall difference between the four materials (F₃,₃₆ = 56.8; *p* < 0.001; η^2^ = 0.83). Mean (± SD) peak loads were 3148.4 ± 643.4 N for BioFlx, 2703.4 ± 243.1 N for SSC, 1076.1 ± 186.9 N for NuSmile and 565.5 ± 159.6 N for ProfZr (Table 2, Fig. 2). According to Tukey HSD post-hoc analysis, BioFlx showed higher fracture loads than both zirconia groups (*p* < 0.001 for either) while the BioFlx–SSC difference did not reach statistical significance (*p* = 0.093). SSC also exceeded both zirconia groups (*p* < 0.001 for either). Within the zirconia groups, NuSmile showed higher fracture loads than ProfZr (*p* = 0.043).

**Table 2:** Fracture loads (N) of the tested crowns and Tukey HSD pairwise comparisons (α = 0.05)

Crown type	Mean ± SD (N)	Min	Max	vs BioFlx	vs SSC	vs NuSmile	vs ProfZr
BioFlx	3148.4 ± 643.4	1835.3	4070.36	—	0.093	< 0.001***	< 0.001***
SSC	2703.4 ± 243.1	2445.01	3210.31	0.093	—	< 0.001***	< 0.001***
NuSmile	1076.1 ± 186.9	747.46	1325.89	< 0.001***	< 0.001***	—	0.043*
ProfZr	565.5 ± 159.6	367.24	808.32	< 0.001***	< 0.001***	0.043*	—

Data are mean ± standard deviation. Overall one-way ANOVA *p* < 0.001 (η^2^ = 0.83). Pairwise comparisons by Tukey HSD

*p < 0.05; **p < 0.01; ***p < 0.001

Failure modes differed by material. Both zirconia groups showed mainly cohesive failures within the crown material (NuSmile: 5/10; ProfZr: 6/10). Mixed failures were most frequently observed in the SSC group (8/10). In the BioFlx group, mixed and adhesive failures were the most common patterns, observed in 5/10 and 4/10 specimens, respectively (Table 3). The Fisher exact test showed that the failure-mode distribution differed between materials (*p* < 0.01).

**Table 3:** Distribution of failure modes after fracture testing (*n* = 10 per material)

Crown type	Cohesive — n (%)	Adhesive — n (%)	Mixed — n (%)	Total
BioFlx	1 (10)	4 (40)	5 (50)	10
SSC	0 (0)	2 (20)	8 (80)	10
NuSmile	5 (50)	2 (20)	3 (30)	10
ProfZr	6 (60)	2 (20)	2 (20)	10

Cohesive = fracture within the crown wall; adhesive = separation at the cement–crown interface; mixed = combination of cohesive and adhesive patterns, scored at × 20 stereomicroscopy. The distribution differed between materials (Fisher exact, *p* < 0.01)

## Discussion

The present in vitro study compared post-aging occlusal wear and fracture load among four prefabricated crown systems using a standardized laboratory protocol. The null hypothesis was rejected for both outcomes. Zirconia crowns showed lower wear scores, whereas BioFlx and SSC showed higher fracture loads than the zirconia groups. These findings show that visible wear resistance and fracture load should be interpreted as separate mechanical properties rather than as a single measure of overall crown performance.

The low wear scores of both zirconia products are in line with reports that monolithic zirconia ceramics resist volumetric loss under cyclic loading better than polymer or resin-based crowns [23]. The glazed/polished surface acts as a barrier to micro-scratch propagation and crack initiation, so progressive wear is limited [24]. This may partly explain why the zirconia groups showed lower fracture loads and more cohesive failures in the present study.

BioFlx produced the highest occlusal wear in our cohort. This is compatible with earlier reports of higher volumetric loss in BioFlx than in zirconia when scored after chewing simulation, with the additional observation that BioFlx tends to spare the antagonist [11, 22]. This finding may be explained by the deformation behavior of the polymer matrix during repeated loading. Such deformation can increase visible occlusal wear, while limiting stress concentration within the crown body [25].

SSCs scored intermediate for wear: better than BioFlx but worse than zirconia. Their metallic structure allows ductile deformation, while repeated contact with the antagonist may still produce surface polishing and shallow wear facets over time. Clinical studies have noted that SSCs produce less enamel wear on opposing teeth than zirconia, presumably because the hardness mismatch with natural enamel is smaller [26].

Although BioFlx and SSC are different materials, their fracture loads did not differ statistically under the conditions of this test. This result may be related to the axial loading setup. Under compressive loading, BioFlx may tolerate force through polymer deformation, whereas SSC may tolerate force through ductile metallic deformation. In both materials, this may allow the crown to withstand high loads before failure, and previous comparative studies have reported similar fracture-load trends for BioFlx and SSC [12, 16, 20, 27]. The failure pattern also supports this interpretation. BioFlx showed more mixed and adhesive failures than cohesive failures, suggesting that the crown–cement interface contributed to load distribution. However, this finding should not be interpreted as material equivalence. Different loading directions, indenter geometries, or cyclic loading histories may produce different material rankings [28]. Inter-brand differences among zirconia crowns may also reflect differences in translucency, grain size, sintering protocol, and related changes in strength and toughness [29, 30].

Clinically, all crown systems in this study showed fracture loads above the pediatric bite-force ranges reported in the literature [31–33]. This suggests that fracture under routine masticatory loading may be unlikely in the short term under the tested conditions. However, this finding should not be interpreted as evidence that all materials would perform similarly in the oral environment. Fracture load represents only one aspect of crown performance, whereas clinical durability is also influenced by wear behavior, esthetics, tooth preparation requirements, marginal adaptation, plaque accumulation, gingival response, and patient-related factors such as occlusal habits. Therefore, the present findings should be viewed as comparative mechanical data that may inform material selection, but they should not replace long-term clinical evidence.

The present protocol has several strengths. Wear and fracture testing were performed in independent specimen cohorts, which avoided the influence of chewing-simulation fatigue on the fracture endpoint. All crowns were tested on identical CAD/CAM resin dies and cemented with the same luting cement, reducing substrate- and cement-related variability. In addition, wear scoring was performed by blinded examiners with high inter-rater agreement, and the statistical analysis included effect-size estimates in addition to p values.

Several limitations should be considered when interpreting these findings. First, the chewing simulator applied a near-axial load at a single point in the fossa; intraorally, occlusal loads are oblique, multiaxial and vary in magnitude across the chewing cycle [28]. Second, the aging regimen was intended to simulate approximately one year of clinical service [19]; longer hydrolytic aging, water sorption, pH cycling and enzymatic attack are not captured. Third, the substrate was a printed polymer resin rather than natural dentin. This approach reduced anatomical variability and provided standardized support during loading; however, the resin substrate does not fully reproduce the mechanical and biological complexity of a natural tooth, including dentinal tubule structure, moisture content, periodontal ligament support, and physiological load transfer. Therefore, the absolute fracture-load values should be interpreted as laboratory measurements rather than direct clinical thresholds [34]. Fourth, only one indenter geometry and one cementation protocol were tested; fabrication/processing variables and cyclic loading history can materially shift fracture-resistance outcomes [35]. Fifth, biological responses such as gingival reaction, plaque accumulation, periodontal status, pulpal effects and biofilm behavior were outside the scope of the protocol and remain unmeasured — these factors weigh heavily on clinical material choice and cannot be inferred from mechanical performance [7, 26, 36]. Finally, these laboratory findings require clinical confirmation over longer observation periods before they can be translated into treatment recommendations.

In the present study, zirconia, polymer-based and stainless steel crown systems were tested under the same thermomechanical protocol, with separate specimen cohorts used for wear and fracture testing. This approach helped keep specimen geometry, cementation and outcome assessment consistent across groups. The polymer-based crown system showed fracture loads close to those of SSC, but it also showed greater visible wear than the zirconia crown systems. These findings indicate that wear resistance and fracture resistance should be considered separately when evaluating pediatric crown materials.

## Conclusions

Within the limitations of this in vitro design, the four prefabricated crown systems differed quantitatively in both pre-specified outcomes. The zirconia crown systems produced the lowest occlusal wear scores, whereas the polymer-based and stainless steel crown systems produced the highest fracture loads. None of the tested crown systems demonstrated superiority across both evaluated outcomes. These laboratory findings provide comparative mechanical data, but they do not directly support clinical recommendations. They should be confirmed in long-term clinical studies, because biofilm formation, hydrolytic aging, multidirectional loading and tissue response were not reproduced in this laboratory model.

### Clinical significance

- BioFlx showed high fracture loads under axial compression, with values that did not differ statistically from SSC in this laboratory model.
- Zirconia surfaces resisted wear better than the two non-ceramic options, but their fracture loads under axial compression were lower than those of BioFlx and SSC; clinical material choice should weigh both findings.
- SSC showed high fracture loads and intermediate wear scores in this laboratory model. Therefore, it remains a useful mechanical comparator, although material selection should also consider clinical evidence, biological response, esthetic demands and patient-related factors.

## Supplementary Information

Supplementary Material 1.

## Data Availability

The datasets generated and/or analysed during the current study are available from the corresponding author on reasonable request.
