# Supplementary material for: Wear resistance and fracture strength of BioFlx, zirconia and stainless steel crowns for primary molars after thermomechanical aging: an in vitro comparative study
Source: BMC Oral Health. 2026 Jun 23;26:1672. doi: 10.1186/s12903-026-08912-4 (PMC13548474; doi:10.1186/s12903-026-08912-4)
Supplement: Supplementary file 1 [file 12903_2026_8912_MOESM1_ESM.docx]

**Figure 1.** Dual-function thermocycling and chewing simulation unit

Thermocycling and chewing simulation were simultaneously performed using a dual-function thermocycling and chewing simulation unit to replicate intraoral conditions. Specimens underwent 10,000 thermal cycles between 5 °C and 55 °C and 150,000 chewing cycles at a 50 N load, corresponding to approximately one year of clinical function for primary molars.

**Figure 2.** Fracture resistance testing setup

Fracture resistance was evaluated using a universal testing machine (Instron, Model 2710-003, USA).
